# Guidelines on the diagnosis, clinical assessments, treatment and management for CLN2 disease patients

**DOI:** 10.1186/s13023-021-01813-5

**Published:** 2021-04-21

**Authors:** Sara E. Mole, Angela Schulz, Eben Badoe, Samuel F. Berkovic, Emily C. de Los Reyes, Simon Dulz, Paul Gissen, Norberto Guelbert, Charles M. Lourenco, Heather L. Mason, Jonathan W. Mink, Noreen Murphy, Miriam Nickel, Joffre E. Olaya, Maurizio Scarpa, Ingrid E. Scheffer, Alessandro Simonati, Nicola Specchio, Ina Von Löbbecke, Raymond Y. Wang, Ruth E. Williams

**Affiliations:** 1 University College London, London, UK; 2 Universitätsklinikum Hamburg-Eppendorf, Hamburg, Germany; 3 Korle Bu Teaching Hospital, University of Ghana Medical School, Accra, Ghana; 4 Austin Health Victoria, University of Melbourne, Heidelberg, VIC Australia; 5 Nationwide Children’s Hospital, Columbus, OH USA; 6 Hospital de Niños de La Santísima Trinidad, Cordoba, Argentina; 7 Universidade de São Paulo Faculdade de Medicina de Ribeirão Preto, Riberirao Preto, Brazil; 8 Coufetery Comms, Lapenne, France; 9 Golisano Childrens’ Hospital, University of Rochester Medical Center, Rochester, NY USA; 10 Batten Disease Support and Research Association (BDSRA), Columbus, OH USA; 11 Children’s Hospital of Orange County, Orange County, CA USA; 12 Regional Coordinating Center for Rare Diseases, University Hospital Udine, Udine, Italy; 13 Department of Surgery, Dentistry, Paediatrics and Gynaecology, University of Verona School of Medicine, Verona, Italy; 14 Ospedale Pediatrico Bambino Gesù, Rome, Italy; 15 Practice for Child Physiotherapy, Hamburg, Germany; 16 Evelina, London Children’s Hospital, London, UK; 17 NIHR Great Ormond Street Hospital Biomedical Research Centre, London, UK; 18 Royal Children’s Hospital, Florey and Murdoch Children’s Research Institutes, Melbourne, Australia

**Keywords:** Expert mapping, Guideline development program, CLN2, Batten, Neurodegenerative disorder, Key Opinion Leader, Modified-Delphi

## Abstract

**Background:**

CLN2 disease (Neuronal Ceroid Lipofuscinosis Type 2) is an ultra-rare, neurodegenerative lysosomal storage disease, caused by an enzyme deficiency of tripeptidyl peptidase 1 (TPP1). Lack of disease awareness and the non-specificity of presenting symptoms often leads to delayed diagnosis. These guidelines provide robust evidence-based, expert-agreed recommendations on the risks/benefits of disease-modifying treatments and the medical interventions used to manage this condition.

**Methods:**

An expert mapping tool process was developed ranking multidisciplinary professionals, with knowledge of CLN2 disease, diagnostic or management experience of CLN2 disease, or family support professionals. Individuals were sequentially approached to identify two chairs, ensuring that the process was transparent and unbiased. A systematic literature review of published evidence using Preferred Reporting Items for Systematic Reviews and Meta-Analyses (PRISMA) guidance was independently and simultaneously conducted to develop key statements based upon the strength of the publications. Clinical care statements formed the basis of an international modified Delphi consensus determination process using the virtual meeting (Within3) online platform which requested experts to agree or disagree with any changes. Statements reaching the consensus mark became the guiding statements within this manuscript, which were subsequently assessed against the Appraisal of Guidelines for Research and Evaluation (AGREEII) criteria.

**Results:**

Twenty-one international experts from 7 different specialities, including a patient advocate, were identified. Fifty-three guideline statements were developed covering 13 domains: General Description and Statements, Diagnostics, Clinical Recommendations and Management, Assessments, Interventions and Treatment, Additional Care Considerations, Social Care Considerations, Pain Management, Epilepsy / Seizures, Nutritional Care Interventions, Respiratory Health, Sleep and Rest, and End of Life Care. Consensus was reached after a single round of voting, with one exception which was revised, and agreed by 100% of the SC and achieved 80% consensus in the second voting round. The overall AGREE II assessment score obtained for the development of the guidelines was 5.7 (where 1 represents the lowest quality, and 7 represents the highest quality).

**Conclusion:**

This program provides robust evidence- and consensus-driven guidelines that can be used by all healthcare professionals involved in the management of patients with CLN2 disease and other neurodegenerative disorders. This addresses the clinical need to complement other information available.

**Supplementary Information:**

The online version contains supplementary material available at 10.1186/s13023-021-01813-5.

## Background

CLN2 disease comes under the umbrella of the Neuronal Ceroid Lipofuscinoses (collectively referred to as Batten disease), or historically and specific to CLN2 disease, Jansky–Bielschowsky disease. These are a clinically and genetically heterogeneous group of neurodegenerative disorders, with the age of onset predominantly in childhood [1].

Epidemiological data across all the NCLs are difficult to interpret. NCLs are classified according to the underlying gene defect, which may share similar clinical features of visual loss, seizures, loss of motor and cognitive function, and early death [2]. CLN2 disease previously referred to as late-infantile neuronal ceroid lipofuscinosis (LINCL) (OMIM # 204500) due to its usual presentation, is an autosomal recessive disorder, caused by pathogenic variants in the *TPP1* gene on chromosome 11p15 (EC 3.4.14.9). Incidence and prevalence of CLN2 disease are poorly reported in the literature with one reference quoting 6–8 cases per 100,000 live births [3], although geographical variation occurs [2, 4, 5]. Mutations associated with CLN2 disease include splice-junction mutations, missense mutations, nonsense mutations, small deletions and single-nucleotide insertions [6]. This results in either reduced activity or inactivation of the lysosomal enzyme tripeptidyl peptidase 1 (TPP1) [7], causing the accumulation of ceroid lipofuscin in the lysosomes, massive glial activation and neuronal loss [8]. Ultrastructural analysis of lysosomal storage in CLN2 disease reveals a typical curvilinear profile pattern [9, 10]. The expression of *TPP1* is developmentally controlled, reaching peak expression at 2–4 years of age, when the onset of signs and symptoms of late infantile neuronal ceroid lipofuscinosis (CLN2, LINCL) typically manifest [11]. Early symptoms include new-onset seizures and ataxia, typically in combination with a history of language delay [12].

To confirm a clinical suspicion of CLN2 disease, the recommended gold standard for laboratory diagnosis is the demonstration of deficient TPP1 enzyme activity (in leukocytes, fibroblasts, or dried blood spots) and the identification of pathogenic variants in both alleles of the TPP1/CLN2 gene [13]. When it is not possible to perform both analyses, either demonstration of deficient TPP1 enzyme activity in leukocytes or fibroblasts, or detection of two pathogenic variants *in trans* is diagnostic for CLN2 disease [12]. Limited access to resources in certain regions can lead to a complex diagnostic journey [12]. This causes disjointed care and treatment delay for patients. In the recent past, disease management has been to treat symptomatically and palliatively [7]. However, the enzyme replacement therapy cerliponase alpha (Brineura®, BioMarin Pharmaceutical Inc.) was approved by the FDA and EMA in 2017, following efficacy in attenuating the progression of disease in affected children [14]. Further clinical trials are monitoring its continued effectiveness as well as efficacy in younger pre-symptomatic children. Faster diagnosis may allow children to be treated earlier in the disease or even pre-symptomatically. An early diagnosis also allows timely genetic advice to be offered to the family.

Internationally agreed guidelines, supported by an expert faculty, formed by robust methodology and assessed by independent assessors, are essential. “Clinical Practice Guidelines serve as a great equaliser in the field of rare diseases: as a matter of fact, they can mean the difference between no care/substandard care and patients living longer, healthier lives with fewer complications” [15].

### Health questions to be answered by these guidelines

The five main health questions that these guidelines seek to answer are;

1. How can early identification and diagnostics for patients affected by CLN2 disease be improved?
2. How can the common manifestations encountered by patients and their families affected by CLN2 be improved?
3. Which supportive therapeutic options are currently available, and what is the expert consensus on their appropriate use?
4. Which disease modifying therapeutic options are currently available, and what is the expert consensus on their appropriate use?
5. What are the current knowledge gaps facing clinicians and families affected by CLN2 disease?

### Objectives

Although recommendations for treating and managing CLN2 disease are available, the methodology used to formulate these clinical recommendations has come under increased scrutiny, highlighting the need for robust, independent guidance on the risks/benefits of disease-modifying treatments and the medical interventions used to manage this condition in the context of worldwide prevalence. The purpose of these guidelines is to provide comprehensive guidance for the identification and clinical management of patients with CLN2, independent of age and disease severity. This programme provides vigorous evidence-based and expert-agreed practical recommendations to address the real clinical need for timely diagnosis, management and treatment of patients with CLN2 disease. A validated modified-Delphi methodology was used which complemented other published information available. Two experts (Co-chairs) were selected to lead the project via the expert mapping tool described. This tool identified rare disease experts from across the globe who were approached to lead on a guideline development program for CLN2 disease. The anticipated benefit of this tool is that it may be utilised for other rare disorders, identifying the most appropriate experts to lead guideline development programs, removing selection bias with simple methodology.

The steps are time-consuming and not easy to accomplish. However, the need for finding these experts in this field is crucial. Key Opinion Leader (KOL) tools are not frequently published, and the methodology is infrequently shared.

The guideline program was led by an independent multidisciplinary Steering Committee (SC), recommended by the co-chairs.

All outputs and recommendations were independent of external stakeholder influence. The driving role of the SC was to validate the program process, inform on the objectives, lead the development of questions, and make practical recommendations that can be readily translated to benefit local clinical practice. These guidelines are intended for use by healthcare professionals who manage the holistic care of patients; with the intention to improve disease awareness, clinical outcomes and enhance patient quality of life. In addition, they are intended to be held by families who will enable non-expert health care providers to become aware of CLN2 disease, further empowering all parties to support the management of individual patients.

### Methods and process

#### Expert mapping tool development

In order for the expert mapping tool to identify such experts, a four-stage process was pursued: Relevant Publication Experience, Author’s H-Index, Patient Organisation Event attendance as a chair or speaker, and Scientific Event attendance as a chair or speaker. Each stage was followed methodically and consecutively to ensure reproducibility.

The first stage is designed to capture the highest level of input into the current literature for CLN2 disease. PubMed database was interrogated using predetermined search terms, and resulting publications were subsequently screened for relevance to CLN2. Selected publications were tracked, and all listed authors were sorted according to the number of appearances. Authors who appeared in two or more publications were selected and ranked as follows: two or more publications (score 1), 3–5 publications (score 2), 6–9 publications (score 4) and > 10 publications (score 5).

The second stage determines the authors H-index [16]. Any search engine such as SCOPUS, Publish or Perish, or Google Scholar can be used, although the same engine must be used for all investigators throughout the process, for consistency. Authors were searched by last name, and first name and their profiles were found based on their occupation, middle initial, city and country of residence. The H-index result for each author was ranked as < 30 (score 3), or > 30 (score 9).

Stage three identifies individuals perceived as leading experts among families and advocates. An online search of publicly available information was conducted for experts who have been involved in patient organisation events or conference programmes during the previous 5-years. Search terms were CLN2, Batten disease and Neuronal Ceroid Lipofuscinosis. All chairs and oral presenters were recorded and cross-referenced with the Batten Disease Family Association, UK (BDFA), Batten Disease Support and Research Association, USA (BDSRA) and other CLN2 focused organisations. All oral presenters, poster presentations and chairs were tallied and ranked as either < 3 appearances (score 6), or > 3 appearances (score 9).

The final stage reveals those in the field perceived as experts by their peers and whose expertise is most called upon by the medical community to present their knowledge on CLN2 disease. A search was conducted for all scientific meetings that had a relevance to, or overlapping focus to CLN2, during the previous 5 years (Additional file 1: Appendix 1). Event programmes were reviewed to identify the speakers and chairs relating to NCL disorders. The number of appearances were recorded and ranked as 2–5 appearances (score 2), 6–9 appearances (score 4) and > 9 appearances (score 6).

The weighted scores of each of the four stages were totalled, creating a new ranking of experts. Those who did not feature in multiple rounds or those with a ranking score of < 10 were not considered. Within the final list, animal experts and industry professionals were not considered for chair positions. To remove bias, both currently practising and retired clinicians who have worked directly with CLN2 patients were included. Also, in order to remove bias, drive international collaboration and fill knowledge gaps, it was proposed that one chair was from Europe and the other from the rest of the world. The exception to this criterion was to review the list of leading clinicians, and once the geographic contrast was exhausted, all the experts on the list were considered in order of total score, regardless of geographical location. Full data analysis for the Expert Mapping Tool has been presented elsewhere [17] (Additional file 1: Appendix 2).

#### Convening the Steering Committee

The expert mapping tool identified two co-chairs, who then debated over which specialities were needed to best encompass all aspects of disease management. The SC was then selected based on their long-standing patient organisation involvement, combined with their academic output, covering the entire scope of the guidelines.

A list of 21 Steering Committee experts from Argentina, Australia, Brazil, Germany, Ghana, Italy, United Kingdom and the U.S.A., contributed to the guideline development. These experts comprised seven specialities: geneticists, paediatric neurologists, neurosurgeons, paediatricians, nurses, physiotherapists, epileptologists, and one patient advocate from the Batten Disease Support and Research Association (BDSRA). The SC was led by the two co-chairs, who advised and drove the program and advocated the program. Further details, including the competing interests, institutions and contribution of each SC member are listed within the declarations section of this manuscript.

#### Systematic literature review methodology

Running parallel to the expert mapping tool and SC selection, two systematic literature reviews were independently conducted by one internal, and one contracted medical writer, focusing on accumulating current evidence for treatment and management of CLN2 disease. Results were reported in accordance with the Preferred Reporting Items for Systematic Reviews and Meta-Analyses (PRISMA) statement [18]. Each literature search was performed in February and March 2019, respectively, through the Google Chrome browser, using PubMed and Google Scholar, and interrogation of internal CBD literature libraries. Both searches were set to the same search criteria to include: publications since 1970, both human and animal trials, grey literature, and full-text. English articles only were selected as the experts noted from recent literature reviews that the articles published in other languages around CLN2 are few and that it was also difficult to distinguish CLN2 from other NCL disorders. Search strings incorporated Medical Subject Headings (MeSH): ceroid lipofuscinosis-neuronal and late infantile ceroid lipofuscinosis, Batten disease, Jansky–Bielschowsky. Free text keywords were defined based on Problem/Patient/Population, Intervention/Indicator, Comparison, Outcome (P.I.C.O.) methodology [19–21] to answer each of the clinical questions (Additional file 1: Appendix 3). The types of literature included were: systematic reviews, meta-analyses, reviews, chart reviews, descriptive observational studies (such as case reports, case series, patient registry data), and interventional early phase non-randomised and open-label clinical trials. Bibliographies of identified publications and reviews were checked for additional relevant studies, and all steering committee members were invited to provide publications that the literature reviews had not retrieved. The extracted details from included articles were: general focus (genetics, diagnostics, clinical management, therapy), study design, patient population, intervention or exposure, comparison (if applicable), outcomes and limitations. Publications were excluded if they were not specifically related to CLN2 disease, animal studies, preclinical studies and single case reports (full exclusion list Additional file 1: Appendix 4). One co-chair (AS) wrote the first draft of the clinical care statements, which were subsequently used for the first consensus-building meeting. The credible link between these publications and the draft statements was verified by an internal medical writer, who used the Oxford Centre for Evidence-Based Medicine (OCEBM) criteria [22, 23] to assess each piece of literature that was linked to each existing statement and assigned a grade (Additional file 1: Appendix 5). One co-chair (SM) also independently assigned an OCEBM grade to each publication and cross-referenced the grades to those assigned by the internal medical writer. A member of the SC was appointed by this co-chair to independently assign OCEBM grades to the literature and review the grades previously assigned. Both the co-chair and the SC member independently identified any articles or evidence that was missing from each statement. The average grades for each article were taken, and then the ratings of each article which related to each statement were averaged to give an overall evidence grade for each statement.

#### Consensus building: statement development meeting.

Steering committee members were invited to join an initial virtual meeting hosted on the Within3 online hosting platform [24], which was used to establish the SC’s communication preferences, biographies, conflict of interest statements, their availability during the programme and suggestions for further committee members. Within3 is a virtual 24/7 environment allowing stakeholders to interact on their own schedule while allowing chairpersons to organise materials in one secure place, post feedback and answer questions. The platform includes an archiving facility.

In a second Within3 meeting the drafted statements were uploaded for the consensus-building phase of the guideline development program. The Within3 session has required resources which, in order to satisfy the requirements of the virtual meeting, must be opened and reviewed.

Medical guidelines have recently been developed which used literature to drive the main topics; these have subsequently been adopted in these guideline statements [25]. The topics in those guidelines, the strength of our updated systematic literature reviews, and the invitation for each steering committee member to add any topics they felt should also be included or exclude any topics, ensured that current evidence was included and that no relevant topic was omitted. This is especially pertinent in rare disease disorders where research is scarce.

A text bar was provided to enable the experts to record their recommended changes to each statement. The survey session was open for 19 days to allow the entire SC the opportunity to participate and respond to the feedback of others. Reminders were sent out during the 19 days to encourage continued activity.

#### Modified Delphi Questionnaire: Health care professionals

Guideline statements resulting from the modified-Delphi questionnaire were systematically validated through an anonymous voting process on Surveylet, a collaborative research software by Calibrum (https://calibrum.com). Healthcare professionals (HCPs) recommended by the SC were invited to participate through a live link and online survey. This process collected the perspectives of all relevant HCPs. In the first round of voting HCPs were asked: whether they had ever managed a CLN2 patient, if so, how many; whether their understanding of the English language was sufficient to complete the survey; their primary role, their main area of expertise, length of time in practice, type of primary practice and in which country. Experts were also asked if they had previously been involved in guideline development groups. The goal was to collect over 60 responses from at least six different specialities responsible for managing patients with CLN2 disease.

The HCPs were asked to validate the guideline statements based on the CLN2 community expert consensus. Each guideline statement was graded via Likert-type scale 1–10, where 1 totally disagreed, and 10 strongly agreed. Consensus was taken at ≥ 75% agreement or more on each statement as the most commonly reported definition of consensus for Delphi studies is per cent agreement, with 75% being the median threshold to define consensus [26, 27].

Where consensus was not reached, the statement was revised by the SC chairs as they felt some simple semantic changes were all that was required to resolve this. If a statement received polarising views, a subject matter expert was invited to present on the topic. Statements that reached the consensus mark were included in this manuscript and were not changed. The survey was left open for eight weeks to collect as many responses as possible.

#### Quality assessment

The AGREEII instrument was used [28] by two independent reviewers to assess the quality of the guideline development strategy and reporting. This validated tool consists of 23 items divided into six domains: Scope and Purpose, Stakeholder Involvement, Rigour of Development, Clarity of Presentation, Applicability and Editorial Independence. Each item is rated on a scale from one (criteria not met) to seven (criteria fully met). Suggested amendments were made where possible; a subsequent second round of review was conducted, and the average of the two review rounds reported. Combined scores for each domain were calculated using the following equation (obtained score-minimum possible score)/(maximum possible score) × 100. An overall average score was calculated from a maximum value of 7.

## Results

### Expert mapping tool

The expert mapping tool identified 1454 professionals, who were sequentially approached after ranking until two were able to commit to participation in this project as chairs. Although the expert mapping tool recommends that one chair should be European and another from the rest of the world, on this occasion, due to the availability of experts, it was not possible. The expert mapping literature review resulted in 155 relevant publications, with 717 published authors, of whom 124 were scored. The highest scored expert was 21, and the lowest was 1. The two selected co-chairs had individual scores of 20 and 16.

### Systematic literature review

The systematic literature review conducted by the contracted medical writer identified 4122 publications. Following the removal of duplicates and exclusion criteria applied, 350 publications were screened and 160 further excluded. Qualitative analysis and PICO summaries were completed for 190 papers. The systematic literature review conducted by the internal medical writer identified 11,996 publications. Following the removal of duplicates and exclusion criteria, 342 were screened and 158 further excluded. Qualitative analysis and PICO summaries were completed for 184 papers. (Additional file 1: Appendix 4, a,b, respectively).

#### Consensus building: statement development meeting.

During the initial Within3 [24] stage of the consensus process, the steering committee posted over 1200 comments, and the collaborative discussion garnered alignment on the clinical care statements. The SC actively participated and responded to statements, which were reduced from 73 to 53 final clinical care statements. Members gravitated from opposing views to a shared perspective, leading to 53 revised statements created using the majority perspective.

#### Consensus building: Modified Delphi Questionnaire

Of the 41 experts who responded to the questionnaire, consensus ranged between 82 and 98%. 100% had managed a CLN2 patient, and they all considered that their level of English was sufficient to complete the survey. The lowest number of respondents for any question was 35. Physicians made up 93% of the respondents, followed by nurse practitioners, physiotherapists and occupational therapists. Areas of expertise included: paediatric neurologists (54%), metabolic specialists (20%), geneticists (9%), neurosurgeons (2%), paediatricians (6%) and others (9%). While there was a good geographical spread of responses, 20% resided in the U.S.A. (Additional file 1: Appendix 6). Over 78% of respondents had been in clinical practice for over ten years, and over 97% were working in a large referral centre or academic hospital. When asked how many patients they had managed, 54% responded between 0–5, 20% 6–10, and 26% > 10, resulting in a wide range of experience managing CLN2 disease patients. There was no consensus on whether the experts had previously been involved in guideline development, although 20% had no previous experience, 74% had been involved on 1–4 occasions, and only 6% were experienced. Of the 53 statements reviewed, 98% of the statements achieved consensus in the first round. Of the single statement that did not reach consensus, the Chairs revised the statement and launched the Modified Delphi 2 (second round) questionnaire. During this process, 100% of the statements met the consensus benchmarks and were included in the guidelines.

#### Appraisal of Guidelines for Research and Evaluation (AGREE) II Assessment

The SC recommended other health care professionals (HCPs) who were independent of the process, to review the manuscript and identify gaps or areas of confusion.

Two external independent reviewers rated the methodology against each the Appraisal of Guidelines for Research and Evaluation (AGREEII) criteria [28]. In each of the six domains, a percentage of 50% or higher was obtained. Individual scores were 83.3% for Scope and Purpose, 81.0% for Stakeholder Involvement, 65.2% for Rigour of Development, 83.3% for Clarity of Presentation, 50% for Applicability, (which resulted in a lower score due to one question being deemed not applicable, and therefore scored 1) and 78.6% for Editorial Independence. The guidance documents were given an overall assessment score of 5.93 (Additional file 1: Appendix 7).

#### Guideline statements

Guideline statements were developed from the results of the systematic literature review as a starting reference, which revealed 13 different topics of clinical focus. The topics included: General Descriptions and Statements, Diagnostics, Clinical Recommendations and Management, Interventions and Treatment, Assessments, Social Care Considerations, Pain Management, Epilepsy/Seizures, Nutritional Care Interventions, Respiratory Health, Sleep and Rest, End of Life Care, and Additional Care Considerations.

#### General description of CLN2 disease and statements (Table 1)

**Table 1:** General description of CLN2 disease, statements and consensus data

Statement	Responders	Evidence level	Consensus %
Within CLN2, two forms of disease evolution exist; classical CLN2 is where symptoms start earlier, between the ages of 3 and 5 years and the symptoms evolve faster. While Non-classical CLN2 has a much slower disease evolution and symptoms appear as behavioural disorders, movement disorders and ataxia rather than seizures and blindness	41	C	82
Classical CLN2 disease is currently also known as late infantile ceroid lipofuscinosis (LINCL). The classical term Jansky–Bielschowsky disease has a historical value. Batten disease is the umbrella/category term and should be used to regard to all NCL and for clarity for the individual disorders refer to the associated gene	40	D	82
Several phenotypes exist within the spectrum of TPP1-deficiency-related diseases. While one (classic CLN2 disease) is far more common than the others, there is overlap in care/ treatment and patient support	41	C	82
These Guidelines will cover the whole spectrum of disorders caused by mutations in CLN2/TPP1, including those with phenotypes not typically classed as NCL	25	NA	80

Multiple forms of CLN2 disease exist. In the more common form of the disease patients present with slowing of development and psychomotor regression, language delay and typically followed by epilepsy between the ages of 2 and 4 [29], subsequently developing retinal degeneration and blindness by 5 or 6 years of age [30]. Life expectancy is between 6 and early teenage years [20]. Around 13% of patients have a later symptom onset [31], more protracted or mild disease course sometimes with the absence of epilepsy and preservation of visual function and a longer life expectancy [12, 32]. Genotypes from these atypical patients predict reduced, rather than the absence of TPP1 activity. Alternatively, TPP1 activity may be absent in certain cell types, but residual activity may remain in leukocytes [33]. Reduced TPP1 activity is implicated in other heterogeneous autosomal recessive ataxias such as SCAR7 the phenotype previously described as Type 7 Autosomal Recessive Spinocerebellar Ataxia or other atypical presentations of CLN2 disease [34]; thus the diagnostic workup for unexplained spinocerebellar ataxias should also include analysis of TPP1 enzyme activity.

Clinicians should, where possible, provide every family with detailed diagnostic, biochemical and genetic information. Four statements were developed to support the general description of CLN2.

#### Diagnostics (Table 2)

**Table 2:** Diagnostic statements and consensus data

Statement	Responders	Evidence level	Consensus %
Diagnosis of CLN2 during infancy is critical to optimise patient outcomes which would benefit by newborn screening	41	D	85
Patients with the existence of a significant speech delay or decline, clumsiness and undiagnosed/unattributed epilepsy before the age of 4 should be tested for CLN2 Disease	40	D	92
The diagnosis of CLN2 can be confirmed by low levels of TPP1 enzyme activity and should be double confirmed by detecting two disease-causing mutations in the CLN2 gene	40	C	91
Early diagnosis as soon as possible after or before symptom onset is crucial and is done by biochemical testing following unprovoked seizures and or unsteadiness in children who may also present delay/decline in psychomotor development, including speech delay	40	B	88

The diagnosis and management of CLN2 disease remain ongoing challenges due to low disease awareness, non-specific presenting symptoms and poor access to diagnostic testing in certain regions [5]. Late-infantile CLN2 disease should be considered in young children with delayed acquisition of, or decline in, language and new onset of seizures [35].

Diagnostic methods have evolved considerably over the last 30 years. Traditionally, electron microscopy of muscle [36], skin and conjunctival biopsies [37] provided valuable diagnostic information. Ultrastructural diagnosis by using biopsies requires the support of clinical and electrophysiological findings [38], but for CLN2 disease this has now been superseded by enzyme testing removing the need for these more invasive and lengthy tests. Ultrastructural examination of peripheral blood lymphocytes in the NCLs reveals different specific cytoplasmic inclusions, with curvilinear profiles typically occurring in classic CLN2 disease [39–41], which were used in prenatal diagnosis using electron microscopy [42, 43]. The first successful prenatal test using DNA and enzyme-based methods on amniocytes was reported in the early 2000’s [44], and a little later by enzyme and mutational analysis of first-trimester chorionic villi [45]. Molecular analysis with allele-specific primer extensions can facilitate prenatal diagnosis, where the familial mutation is known [46].

Specific polyclonal antibodies against TPP1 detect the absence or marked reduction of this protein in lymphocytes, lymphoblasts, fibroblasts [47] and brain homogenates [48] from LINCL patients, a technique found to be accurate, cost-effective, and rapid.

Early laboratory diagnostic methods required the support of neuroradiological findings. Marked cerebellar atrophy is visible at an early stage of CLN2 disease [49]. Neurophysiological findings are characteristic for classic late infantile CLN2 disease, with early presentation of a typical paroxysmal spike‐wave response in response to low frequency intermittent photic stimulation (IPS, 1–2 Hz) (the photoparoxysmal response (PPR)) by electroencephalogram (EEG) [50, 51]. This early photosensitivity is a hallmark of CLN2 disease, particularly if accompanied by delayed speech and/or ataxia [52]. Diminished EEG and enhanced cortical visual evoked potentials (VEP) are seen in the later stages of disease [52]. In contrast, vision loss occurs later in the disease of CLN2 that other NCL subtypes and is not a clinical hallmark for diagnosis [53]. International experts had met in 2015 and recommended best laboratory practices for early diagnosis of CLN2 disease [12]. In any family with a hereditary metabolic brain disorders, early or prenatal diagnosis is paramount both for clinical management, maximising the benefit from therapies, and for the adjustments in family lifestyle and future family planning [54]. The genetic heterogeneity in NCLs demonstrates the importance of DNA testing to accurately identify affected individuals and carriers [55].

Four statements were developed to support the current recommendations of diagnostic methods.

#### Clinical Recommendations and Management of CLN2 disease (Table 3)

**Table 3:** Clinical recommendations and management of CLN2, statements and consensus data

Statement	Responders	Evidence level	Consensus
All patients with suspected CLN2 disease should be referred to a centre with expertise in managing patients with NCL disorders	41	NA	95
The first consultation should be conducted by a physician with experience of treating CLN2, when possible, as soon as possible after diagnosis. This should include a full discussion of disease pathology, progression, treatment options and management. Ongoing information should be provided to optimise patient outcomes	41	NA	95
A paediatric neurologist, rare disease specialist with clinical experience in CLN2 disease supported by a local multidisciplinary team, should lead the patient’s care	41	NA	89
Holistic care is critical for CLN2 management and a multidisciplinary team (MDT) is advised where possible to manage the diverse range of disease manifestations	41	B	96
Emotional and psychological family support should be recommended and offered by an appropriate health care provider to the patient, caregiver and full family	41	D	97
Psychological support or counselling should be offered/made available, where available to families following diagnosis and should be informed of relevant patient organisation contacts when deemed appropriate	41	NA	95

Management of CLN2 disease should be guided by the standards and guidelines from the International Children’s Palliative Care Network [56] with a holistic approach to supporting both the patient and their families. This requires a skilled multidisciplinary paediatric team of physicians, nurses and therapists, dieticians, psychologists, social workers and counsellors [25]. Supportive behavioural and symptomatic treatments, including anti-epilepsy medication, are warranted [20, 57]. Six statements were developed to support the clinical and management recommendations.

#### Assessments (Table 4)

**Table 4:** Assessments, statements and consensus data

Statement	Responders	Evidence level	Consensus
In order to monitor the disease progression, it is recommended that a patient receives baseline assessment to track disease progression, a series of tests where possible including EEG, Visual Exam, Epilepsy Record and Medication Utilisations, a record of MRI scans and cognitive testing. These exams focusing on physical and neurological manifestations should be repeated on an interval agreed to by the MDT (6 months or annually)	41	C	84
Currently, two tools are used for disease progression, namely the Hamburg Scale and the Unified Batten Disease Rating Scale, which are most widely used and accepted within the CLN2 Community	38	B	85
A comprehensive medical history and multi-system evaluation should be conducted following diagnosis and at parent and care providers discretion to set a baseline for ongoing assessments and evaluate the physical and neurological manifestations of disease, functional ability and disease burden	40	C	90
Ongoing and regular multi-system monitoring and assessments are recommended to track the natural history of CLN2, monitor the impact of treatment and assess the need for treatment interventions to manage the symptoms of CLN2. These should be conducted at every clinic visit, annually or in some cases as clinically indicated	41	D	90
A physical examination should be performed during every visit to assess general health, growth, vital signs, visual performance, frequency of seizures, developmental assessment and new significant medical events	41	NA	90
MRI of the brain is recommended at diagnosis if not already performed in patients with CLN2 and should be repeated as needed	40	D	89
Age-appropriate evaluations by an ophthalmologist are recommended every 6 months if possible, or at least annually	39	NA	92
Annual or more frequently if needed patient-reported outcomes is recommended to capture disease impact on patients and their families	40	NA	90
Regular therapy and assessments should be provided in a comfortable local setting agreed with the family by physiotherapists and speech therapists and anticipatory/timely provision of supportive devices, as well as regular therapy such as music therapy and other activities that reflect the interest of the patient	37	NA	95

A comprehensive medical history and multisystem evaluation should be conducted at the first clinic visit to evaluate the physical and neurological manifestations of the disease and establish a baseline for natural history assessments. This should include general health, growth, vital signs, age at onset, language and motor difficulties, behavioural abnormalities, seizure frequency, feeding issues, ophthalmic examinations, full neurological evaluation and brain Magnetic Resonance Imaging/Spectroscopy (MRI/MRS) [35, 58]. As part of the initial evaluation, some centres may find EEG with polygraphic recordings useful to detect the photoparoxysmal response [52]. Rating scales for neurological decline and imaging can provide valuable benchmarks for disease progression and severity [59]. Assessments should be ongoing and regular at each clinic visit, or as clinically indicated.

In order to evaluate disease progression clinically, the use of the Hamburg scale [60] is well validated to assess the regression of motor and language function as well as epilepsy and vision. The Weill Cornell LINCL scale is an adapted version of the Hamburg scale and adds the category swallowing and myoclonus [58]. Both scales have been combined and definitions of scores edited in order to be used as efficacy measures in clinical trials.

Volumetric analysis of cortical grey matter loss, volume percentage of cerebral spinal fluid (%CSF) by MRI and, N-acetyl aspartate to creatinine ratios (NAA/Cr) from whole-brain MRS techniques, have been proposed as quantitative biomarkers of disease progression [61, 62].

While the relationship between neurological function and ophthalmic manifestations in CLN2 disease is not well defined, ophthalmic degeneration closely correlates with the degree of neurological function and the age of the patient [53]. Full-field ERG may be useful for NCL diagnosis, particularly for those who do not have access to genotyping [63]. Non-invasive assessment of ongoing macular and retinal degeneration can be performed by using optical coherence tomography (OCT) and quantified over time [53]. Age-appropriate ophthalmology evaluations are recommended every six months, along with patient-reported outcomes annually, or more frequently if necessary. Nine statements were developed to support the recommendations for clinical assessments.

#### Interventions and treatments for CLN2 disease (Table 5)

**Table 5:** Interventions and Treatments for CLN2 disease, statements and consensus data

Statement	Responders	Evidence level	Consensus
Initiation of long-term ERT with cerliponase alfa at 300 mg (or age-appropriate) dose every other week through intraventricular infusion is suggested in non-classical TPP1 deficiency patients after confirmed diagnosis and agreement between parents and provider, as long as no contraindications to therapy exist. Initiation of long-term ERT with cerliponase alfa at 300 mg (or age-appropriate) dose every other week through intraventricular infusion is recommended in classical CLN2 patients with the potential to benefit from this therapy	37	C	84
Disease-modifying treatment with a licensed therapy ideally should be delivered by a team experienced in the management of CLN2 disease and use of any required devices. For current ERT treatment for CLN2 disease, this includes brain intraventricular devices	39	C	93
There is no evidence currently that HSCT benefits patients with CLN2 and at this time is not recommended or approved as a treatment	34	C	93
Intraventricular devices should be placed under general anaesthesia by a very experienced paediatric neurosurgeon	36	C	92
Intraventricular device should only be accessed by a trained individual to limit/ minimise complications	39	C	95

Various treatment strategies are under clinical development for the treatment of NCLs, although to date, there is only one clinically approved drug for CLN2 disease [64]. Recombinant human TPP1 (cerliponase alfa, Brineura™) is an enzyme replacement therapy (ERT) that slows the decline of motor and language function in CLN2 patients [14]. The approval of cerliponase alfa (2017) in the European Union (EU) covers all ages, and the Food and Drug Administration (FDA), for patients of 3 years and above [65]. Clinical trial evidence revealed that the therapy is well tolerated. As in other enzyme replacement therapies, the development of anti-drug antibodies (ADA) represents a constant risk for allergic reactions and—if antibodies are neutralising—for loss of treatment efficacy. Although ADA production was detected in the cerebrospinal fluid (CSF) and serum, of 25% and 79% patients, respectively, this was not associated with neutralising antibodies, any incidence of hypersensitivity adverse reactions, or reduced therapeutic response [66].

Cerliponase alfa is administered every two weeks via slow intracerebroventricular (ICV) infusion (300 mg), using a Huber non-coring needle and syringe pump with post-infusion flushing of the line to ensure complete dosing. This technique requires device implantation under general anaesthesia, by an experienced paediatric neurosurgeon. General anaesthetic comes with an elevated risk of harm in the NCL population [67]. Extreme muscle atrophy, seizures and upper airway obstruction add potential complications and may need to be managed accordingly by specialist anaesthetists. |A risk of significant hypothermia under general anaesthetic is supported by a case study of a 14-year old child with CLN2 disease [68]. A further potential anaesthetic risk to this population involves the pathology of the heart, which includes cardiomyopathy and repolarisation disturbances, described in older patients but which maybe evolving in younger patients [69].

Investigators recognise that combinational therapeutic approaches will be required to tackle the multiple whole body aspects of any NCL [70, 71] Current efforts are aimed at developing therapies that effectively attenuate neurodegeneration in both the brain and the retina [64]. Five statements were developed to support the recommended interventions and treatments.

#### Additional care considerations for CLN2 disease (Table 6)

**Table 6:** Additional Care Considerations for CLN2 disease, statements and consensus data

Statement	Responders	Evidence level	Consensus
CLN2 should be managed holistically by a multidisciplinary team to address and manage all symptoms of the disease	40	D	95

**Fig. 1:**
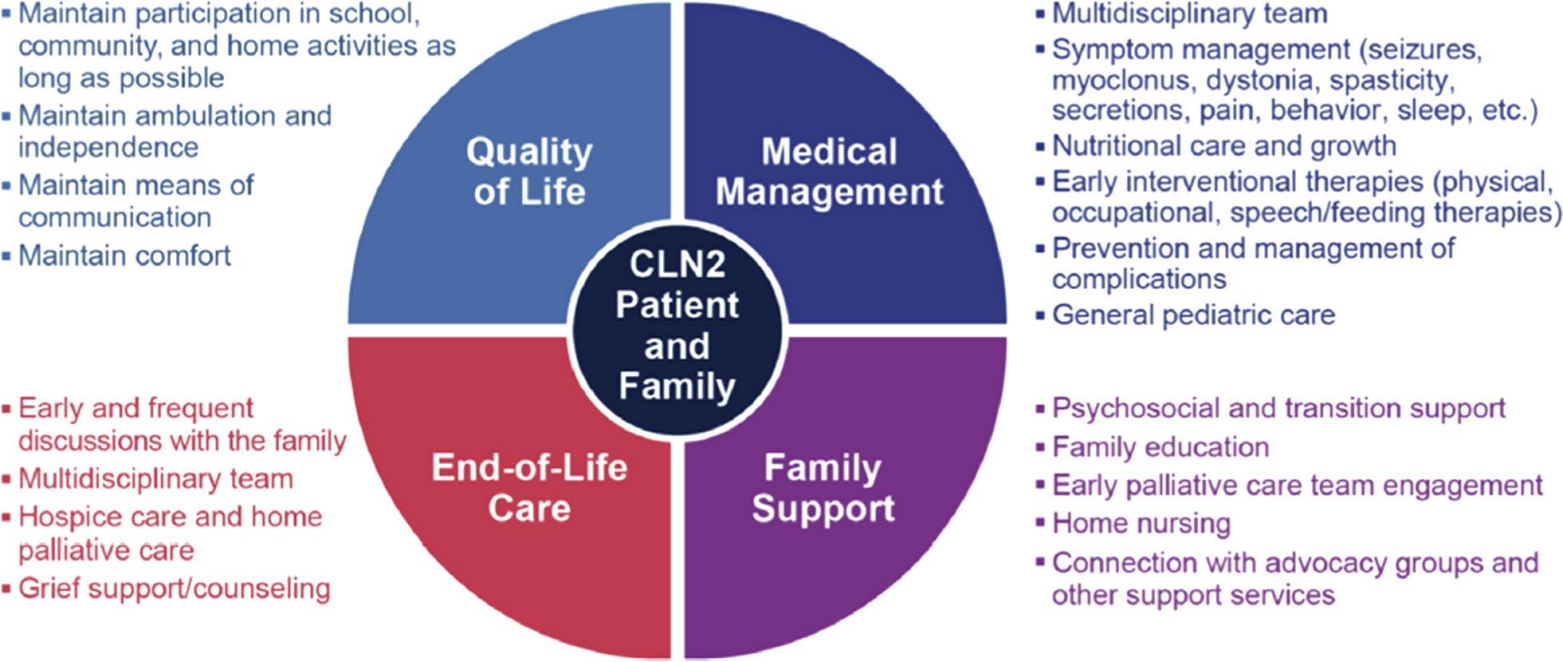
A palliative care framework for CLN2 disease management facilitates comprehensive care of patients and families. Figure taken from Williams et al. [25] Management strategies for CLN2 disease. http://dx.doi.org/10.1016/j.pediatrneurol.2017.01.034. Published by Elsevier Inc. an open access article under the CC BY-NC-ND license (http://creativecommons.org/licenses/by-nc-nd/4.0/)

As classic late infantile CLN2 disease progresses, there is a high symptom load and rapid rate of functional decline. The crucial goal is the maintenance of quality of life, for the patient and their family. Psychosocial support is imperative. A framework should be in place for comprehensive patient and family-centric care, which must evolve as the disease progresses (Fig. 1). Frequency of clinic visits should be tailored to meet the individual needs of the patient and their family. One statement was developed to support the additional care considerations.

#### Social care considerations for CLN2 disease (Table 7)

**Table 7:** Social Care Considerations for CLN2 disease, statements and consensus data

Statement	Responders	Evidence level	Consensus
Adaptations and support for communication is essential and a speech and language expert should be involved in all patients with CLN2	41	NA	92
Considerations should always be made in order to maintain a patient’s activity and social interaction by aiding their mobility, communication abilities, and special considerations around loss of vision	41	NA	95
Considerations to teach patient alternative communication strategies and strategies for utilising audio sense following vision dysfunction may assist a patient in their ability to socialise	40	NA	92
Early use of medical aids such as orthoses, therapy chairs, standing and walking equipment supports mobility which improves quality of life	41	NA	92
Caregiver burden has a significant impact on families affected by CLN2, and appropriate tools should be used to capture this	41	D	92
Visual support is critical to maintaining function, and all measures should be employed to maintain visual ability	41	NA	86
Physical, occupational, speech, and other supporting therapy interventions are recommended for patients in order to maintain activity and the highest of quality of life	41	D	94

CLN2 disease has a profound impact on family life. The physician should be prepared for the reaction from family as the diagnosis often comes after a protracted diagnosis journey of two or more years. At the time of diagnosis, families should be provided with information, relevant resources and encouraged to ask questions. Contact details from patient advocacy groups should be offered to support all family members [20]. Engagement of the palliative care team is essential and must be managed on the basis of individual need, and in line with available resources. Genetic counselling and family planning should be offered. Grief and bereavement support should be ongoing, and memory-making activities encouraged [25].

Caregiver burden has a significant impact on families, and appropriate tools and adaptations should be put in place [72] to cope with the psychological stress, the physical impact from carrying and lifting, social challenges, and financial strain.

Although children with untreated CLN2 disease are typically unable to walk, talk and are visually impaired by the age of 6, hearing is preserved, and it is essential to maintain school attendance for as long as possible and continue social interactions. Good communication networks should be set up between parents, healthcare givers and school staff to create an environment where the child’s needs can be met at school. There should be a shift in the approach to an educative model which focuses on maintenance of functional abilities rather than gaining new ones. Augmentative communication, such as the use of objects of reference and gestures, can be beneficial and should be implemented early [25]. The high prevalence of behavioural symptoms causes distress to families, mainly because they may be indicative of disease progression [73]. Physical, occupational, speech and complementary therapy interventions should be included in the care package [25].

#### Pain management and movement disorder in CLN2 disease (Table 8)

**Table 8:** Management of movement disorder and pain in CLN2 disease, statements and consensus data

Statement	Responders	Evidence level	Consensus
Mobilisation and repositioning can help reduce pain. Medical aids such as a standing device or systems for positioning in bed should be considered	40	C	93
There is a complex movement disorder in CLN2 disease that includes, but is not limited to, dystonia and involuntary muscle movements. Treatment approaches should be developed in cooperation by experts for NCL and movement disorders	40	C	90
In CLN2 complex movement disorder paired with a complex seizure phenotype and myoclonic jerks might mimic pain-like episodes that have a different origin (e.g. agitation, boredom, fear and even happiness) should be managed pro-actively according to the aetiology	40	C	90

Assessing pain in CLN2 children is challenging because of their lack of language. Therefore patients should be assessed carefully for any possible cause for pain and treatment should be specified accordingly: Gastrointestinal problems such as constipation and gastric reflux are frequent sources of pain. Also, urinary retention, skin breakdown, dental problems, hip luxation, bone fractures due to osteoporosis caused by immobility are all sources of pain in CLN2 disease. It is often difficult to distinguish between pain and other sources of discomfort: fear, anxiety, loneliness or boredom [25]. The Batten’s Observational Pain Scale may be useful for parents monitoring their children’s pain at home [74]. Transdermal fentanyl has shown some efficacy in reducing centrally mediated pain [75].

The complex movement disorder phenotype associated with CLN2 disease includes non-epileptic myoclonus as a hallmark as well as dystonia, spasticity, chorea, athetosis, and tremors. These symptoms represent a high disease burden to the patient and are very difficult to control and can be aggravated by pain. Therefore, pain should always be treated first or ruled out. Advice from movement disorder experts is often helpful. Recommended pharmacologic treatment includes benzodiazepines, phenobarbital, levetiracetam, pregabalin, valproate, lamotrigine, and zonisamide [25]. However, these should be selected carefully and periodically re-evaluated. Positioning aids, weighted blankets, physiotherapy, heat and medications may help to ameliorate pain [25]. Discomfort caused by spasticity can be effectively treated with physical therapy, baclofen and tizandine. Three statements were developed to support movement disorder and pain management recommendations.

#### Epilepsy and seizures in CLN2 disease (Table 9)

**Table 9:** Epilepsy and seizures in CLN2, statements and consensus data

Statement	Responders	Evidence level	Consensus
Epilepsy management should include consideration of the most appropriate medications for CLN2 disease and those AEDs that are not recommended	38	C	88
ALL recommended medication to be listed out in a table with a clinically suggested sequence depending on the stage of disease progression	38	NA	88

Multiple seizure types are observed in CLN2 disease, including myoclonic, tonic, atonic, absence and tonic–clonic. The goal of seizure management is to minimise the impact of seizures on the child’s well-being, thereby supporting social interactions, mobility and fall prevention [25]. Epilepsy in children with polymorphic seizures (including CLN2 disease) is largely therapy-resistant [76], and complete seizure freedom is most likely not possible to accomplish even with polytherapy. Certain AEDs such as carbamazepine and phenytoin have been reported to exacerbate myoclonus and should be avoided [20]. Other AEDs may have side effects similar to disease symptoms such as language impairments due to topiramate; therefore, these should be used with caution [25]. While valproate is the most commonly used medication in seizure management, its long term use has also rarely been implicated in the exacerbation of dystonia in late disease stages [25], with associated hyperthermia and hyper creatine kinase (CK)-aemia [77]. As the disease progresses, myoclonic seizures can predominate and are difficult to control [29]. Reports on the application of cannabis in paediatric epileptology have been widely published. The component tetrahydrocannabinol (THC) may reduce spasticity, improve dystonia, increases initiative and interest in their surroundings, and is anticonvulsive [78]. However, more recently, the compound cannabidiol has been shown to have fewer side effects and be more efficacious in NCL patients [79]. To the expert’s knowledge, there is no table of recommended medications depending on the stage of disease progression. While a list of common medications used to treat CLN2 disease symptoms is reported in Williams et al. [25], of which they recommend periodic re-evaluation, further investigation is necessary for the optimisation of medication regimes for seizure management and movement disorder.

Children with CLN2 disease may be prescribed multiple drugs (between 10–12), and there should be an awareness of drug-drug interactions [25]. It is recommended to minimise polypharmacy as far as possible. A ketogenic diet has also been shown to be effective in treating multiple seizure types [80] and in drug-resistant seizures, although patients should be closely monitored for side effects, such as constipation, kidney stones and growth retardation [25].

Seizures in CLN2 disease can be life-threatening, and emergency seizure management plans for home and school should be put in place. Medications for emergency use include buccal or intranasal midazolam, diazepam or lorazepam [25]. Two statements were developed to support the recommendations for epilepsy and seizure management.

#### Nutritional Care Interventions (Table 10)

**Table 10:** Nutritional Care Interventions in CLN2, statements and consensus data

Statement	Responders	Evidence level	Consensus
Any patients affected by CLN2 should be fed according to his/her CNS grade of integrity, or there is no evidence to support feeding CLN2 patients different to any other patient affected by a neurodegenerative disease	38	D	86
For CLN2 patients over age 16 years with significant dysphagia, enteral tube feeding should be considered according to current NICE guidance: https://www.nice.org.uk/guidance/cg32/chapter/1-Guidance#enteral-tube-feeding-in-hospital-and-the-community	35	NA	83
Tube feeding should be considered if one of the following is present: Increased risk of choking, Inability to meet nutritional requirements, Confirmed silent aspiration on video fluoroscopy, Repeated episodes of aspiration pneumonia confirmed by imaging	40	NA	94

Nutritional management is critical to patient care. Swallowing difficulties arise and worsen until oral food intake (eating and drinking) fails to meet nutritional requirements, and there is a high risk of aspiration. Caregivers should be educated on appropriate food consistencies and how to recognise and alert clinicians to early signs of oro-pharyngeal dysfunction. Pharmacological and non-pharmacological interventions can be recommended to manage oral secretions [25]. Therapeutic support for orofacial regulation should commence as soon as swallowing difficulties occur, and maintained during tube feeding to reduce secondary damage. Even if the child has a feeding tube, it remains necessary to be able to swallow saliva and close the mouth. A stepwise program of anticholinergic treatment is necessary to minimise drooling, although side-effects such as constipation and urinary retention may be observed [25]. Regular intermittent, low-dose botulinum toxin injections to the salivary glands, may also help to control symptoms. Tube feeding is recommended when aspiration risk becomes high, and again families should be advised on gastrostomy tube home care and enteral feeding [25]. Three statements were developed to support nutritional care recommendations.

#### Respiratory health (Table 11)

**Table 11:** Respiratory health in CLN2, statements and consensus data

Statement	Responders	Evidence level	Consensus
In CLN2 patients respiratory health contributes to disease burden but can be maintained by supportive measures	41	NA	89
CLN2 children should have all their normal childhood vaccinations or add exclusions	38	D	92
Family members, caregivers or relatives are also urged to vaccinate to lessen the risk of patient viral contraction	39	D	93

Respiratory complications may quickly become life-threatening, especially in the latter stages of CLN2 disease. Vaccinations against preventable respiratory diseases are recommended for the whole family [25]. Interventions such as regular pulmonary hygiene, for example: using mucolytics, high-frequency chest wall oscillation, mechanical insufflator-exsufflator devices and bronchodilators, are recommended [25]. In addition, regular mobilisation in age-appropriate positions, and manual interventions to improve lung function within physiotherapy should take place. Three statements were developed to support respiratory health recommendations.

#### Sleep and rest (Table 12)

**Table 12:** Sleep and rest in CLN2 disease, statements and consensus data

Statement	Responders	Evidence level	Consensus
Insomnia and sleep disturbance are common, and this should be actively monitored and managed as per local practice. Fixed cushions for positioning are recommended to avoid motor restlessness, the risk of swallowing saliva and fear of choking	39	C	90
Maintaining proper sleep is vitally important for the patient and the caregivers; therefore it is important to ensure good and sufficient sleep for the whole family	41	C	95
A patient should be supported as required to continue to engage and socialise at school or other facilities for as long as possible	41	D	96
Living with a rare disease is challenging to the whole family and appropriate support should be offered to caregivers, siblings and family members	40	D	97

Sleep and rest are equally important for the patient and their caregivers. Sleep deprivation due to the need to be constantly on alert for seizures is common for the caregiver. Psychopathological symptoms such as sleep disturbance, fear, aggressive behaviour, depression, and hallucinations are a particular challenge [29]. The Children’s Sleep Habits Questionnaire is a validated tool used for both behaviourally and medically-based problems [81]. The majority of children with CLN2 disease have sleep disturbance [81], which in turn adversely affects seizure control and exacerbates behavioural [73] and cognitive impairments [25]. Behavioural and environmental strategies and medications may be helpful in treating sleep dysfunction [25]. Sleep-disordered breathing was a prominent concern for families, and a polysomnogram is recommended for children with a sleep disturbance and snoring to identify a treatable concern [81]. Melatonin is safely and frequently used for sleep onset difficulties in children with neurodevelopment disorders [82], although its efficacy remains controversial and more evidence is required [83].

Four statements were developed to support the recommendations for sleep and rest management.

#### End of life care (Table 13)

**Table 13:** End of life care for CLN2 disease, statements and consensus data

Statement	Responders	Evidence level	Consensus
Important considerations as nearing ‘end-of-life care’ patient comfort, including reduction of pain and anxiety as well as support for continued activities and interactions, and support for family and caregivers	41	C	98
Palliative care services are important, and a plan should be recorded and offered at the end of life, if or when available	39	D	96

The psychological impact on caregivers whose children have a life-limiting disease is profound, and the palliative care team should be engaged to discuss milestone losses and set expectations (Fig. 1). A palliative care framework for CLN2 disease management facilitates the comprehensive care of patients and their families [25]. Palliative therapies in NCL diseases represent a significant challenge due to multiple symptom complexes and affected body systems [29]. The major goal at the end of life is alleviation of pain and distress. Respiratory comfort can be improved by frequent repositioning and using positioning aids. If possible, the positions should be age-appropriate to achieve the greatest possible participation in everyday life. Hospice and home palliative care services should be offered, although there are regional barriers to the availability of such services [25]. In the later stages of the disease, there should be an emphasis on prevention of secondary complications such as decubitus ulcers, muscle atrophy and aspiration pneumonia [25].

Two statements were developed to support the end of life care recommendations.

## Discussion

Effective management and treatment of CLN2 disease management require an early diagnosis; and therefore, unless there is a familial history, irreversible neurodegeneration has usually occurred before a diagnosis is made [31].

The aim of this programme was to use a robust systematic approach to develop consensus-based guidelines to increase diagnosis rates and raise awareness about the management of symptoms in line with the best available evidence and to aid the development of expected standards of care. The approach (summarised in Addidional file 2) that has been used in this methodology has been used successfully for the development of other medical guidelines; For example, the American College of Medical Genetics (ACMG), interpretation of sequence variants [84] for the diagnosis and treatment of phenylketonuria (PKU) [85], and the Maple Syrup Urine Disease (MSUD) guidelines [86]. It is crucial that the patient and family voice is heard in developing such standards and guidelines.

Our results highlight the critical need for early diagnosis and document the current expected care standards for laboratory, clinical and radiological diagnostic investigations and assessments. Children who present with significant speech delay or decline and clumsiness, without a diagnosis, should be suspected of CLN2 disease, or other lysosomal storage diseases, and should be referred to a specialist centre.

Economic modelling did not form part of this guideline development. However, there may be local cost implications of applying these guidelines which should be considered, and especially in prescribing Brineura. Economic modelling criteria around Brineura were requested from the manufacturer (BioMarin Pharmaceutical Inc.) and from the National Institute for Health and Care Excellence, UK (NICE). These publicly available documents can be found in Additional file 1: Appendix 8, together with a Pharmacoeconomic Review Report: Cerliponase Alfa (Brineura): (BioMarin Pharmaceutical (Canada) Inc.). Other health state-dependent utility values obtained through a utility study conducted in July 2017 that allows for health states to incorporate all relevant aspects of the disease that impact the quality of life, including progressive symptoms that are not captured by the CLN2 Clinical Rating Score are not published but were made available to the steering committee as it was appreciated that costs have significant implications for Health Service decision making around funding and provision of novel therapies.

Start and stop eligibility criteria for enzyme replacement therapies are becoming increasingly important, especially in the rare disease sphere. Looking at all the criteria available in the public domain there are some regional differences, although there is a general agreement. These have been summarised below as a reference (Additional file 1: Appendix 8). These criteria were not part of the guideline development process, regarded as out of scope by the experts due to the variations in health care systems, cultural backgrounds, funding arrangements, facilities and the heterogeneity of the disorder. Further, most of the publicly available stop-start criteria only focus on the classical early-onset form of CLN2 disease. Discussions with the family to review risks, benefits, and criteria for potential initiation/discontinuation of therapy are helpful to clearly communicate expectations of therapy. Quality of life data was not used in the model as they do not represent the full range of CLN2 disease stages or include equivalent data for patients treated with standard care; publications are planned to fill this gap.

These guidelines covers the whole spectrum of the disorder. They all focus on the following main areas: that diagnosis must be confirmed and secure before the onset of therapy, baseline assessment should be performed before the onset of treatment and may include but is not limited to language ability, motor ability, feeding status, seizures, myoclonus and vision. Individual treatment aims are important to be established with the family and accepting that stabilisation of disease at treatment onset may be a very important outcome for some families, as this condition is normally rapidly progressive. Treatment should be discontinued in patients if they are affected by another life-limiting condition, or have infusion-related severe adverse reactions which are not preventable/manageable either by appropriate pre-medication, adjustment of the infusion rate, or other clinical concerns that cannot be resolved.

## Strengths and limitations of the programme

Management of a child affected by CLN2 disease requires a coordinated, multidisciplinary approach. It is, therefore, imperative that guidelines cover a broad range of topics in the clinical management of this disease. The nature of rare diseases often results in a lack of high-quality evidence for medical and treatment interventions. Therefore, each of the consensus statements here was also assessed by the SC chairs, using the Oxford Centre for Evidence-Based Medicine grading system.

While the response rate to each question was high, not all HCPs responded to each question. The reason for this is that the responders were multidisciplinary, and not all questions were relevant to their field of expertise. The multidisciplinary nature of respondents adds strength to the guidelines.

Multiple sponsors funded this programme; however, measures were taken to ensure that this did not influence the final statements (described in the methods section).

The strengths of this programme include the robust methodology, covering the initial selection of expert steering committee and chairs, through to the expert mapping tool, which has previously been presented as a poster [17] (Additional file 1: Appendix 2). This method brought together multidisciplinary experts from around the globe, each contributing not only the knowledge of the condition itself but also local challenges with regards to patient management. The committee included input from a patient advocate. The comprehensive systematic literature review ensured that the guidelines are based on the current evidence base. The use of a modified-Delphi voting process to gain consensus ensured that each of the 53 guideline statements reflected the views of wide-ranging specialists. Risk of bias assessment was not undertaken for these guidelines as the aim is related to the identification of clinical management strategies, diagnoses, holistic multidisciplinary care used by health care professionals. The purpose was not to gather information about the effectiveness of outcomes. The methodology and transparency were also demonstrated via review of the manuscript against the validated AGREE II instrument, where the guidelines gained a score of 5.7 (www.agreetrust.org).

## Future perspectives

Improvements in symptom management and the introduction of ERT, and potentially a future gene therapy for treatment of CLN2 disease is likely to impose new challenges as life expectancy increases. In addition, further investigation is necessary for the optimisation of medication regimes for seizure management and movement disorder. However, due to the availability and variability of drugs in different geographical areas, it was deemed out of scope for this consensus paper. These guidelines aim to refine existing strategies facilitating optimal care to all CLN2 disease patients while taking into account local boundaries. These guidelines were developed to be utilised internationally and as such, can be regarded as the basis for adaptation to local policies. The wide variability in health care systems, geography, economical and cultural differences make it impossible to develop audit guidance acceptable to all. A future aim is to identify and prioritise physicians, nurses and residential care staff via meeting presentations, publications, online-focused multi-audience meetings, and a web-site summarising these recommendations in an easily accessible format, linking to other NCL resources; and to liaise with these providers or stakeholders, including educators and teachers, to ensure consistency. While out of scope for this manuscript, it is the hope of the steering committee that local groups will extract appropriate elements from these guidelines and develop their own implementation and audit cycles. Audited information would be invaluable if it could be fed back for incorporation into the regular reviews and updates to these guidelines.

## Conclusions

This manuscript provides robust evidence- and consensus-driven guidelines that can be used by all healthcare professionals involved in the management of patients with CLN2 disease. The approach that has been taken in the process of these recommendations are also applicable to the symptomatic management of other neurodegenerative diseases. It is recognised that the guidelines provided represent a point in time, and further research is required to address current knowledge and evidence gaps, especially the emergence and effect of new treatments. This manuscript provides one element to the guidelines on the diagnosis, treatment and management of patients with CLN2 disease. It will accompany other resources with plain language summaries and tools to disseminate the information across the medical field. It is intended that the methodology used in these guidelines is robust and will be easily transferred to the development of guidelines for other rare diseases. Ideally, it will be readily accessible online.

The SC recommends that these guidelines are reviewed and updated within five years or sooner if there are significant changes to medical practice; further, to develop criteria to enable monitoring and auditing to assess the implementation of and adherence to the guidelines. The process was led by an independent steering committee and independent of sponsors influence.

This guideline program addresses a clinical need for patients with CLN2 disease and is intended to complement other information available [7, 15, 20, 25, 29, 64], including that of patient support organisations (See Additional file 1: Appendix 9).

## Supplementary Information

**Additional file 1**: Appendices. Appendices 1–9.

**Additional file 2**: Poster. Methodology to develop guidelines for the management of patients with CLN2 disease.

## Data Availability

The datasets used and/or analysed during the current study are available from the corresponding author on reasonable request.
