# Supplementary material for: Guidelines on the diagnosis, clinical assessments, treatment and management for CLN2 disease patients
Source: Orphanet J Rare Dis. 2021 Apr 21;16:185. doi: 10.1186/s13023-021-01813-5 (PMC8059011; doi:10.1186/s13023-021-01813-5)
Supplement: Supplementary file 1 — Additional file 1: Appendices. Appendices 1–9. [file 13023_2021_1813_MOESM1_ESM.docx]

# APPENDICES

## Appendix 1

*List of Scientific Meetings relevant to CLN2 disease during the previous five years:*

- European Paediatric Neurology Society (https://www.epns.info)
- NCL International Conferences (https://www.ucl.ac.uk/ncl-disease/clinicians/ncl-meetings)
- European Study group for Lysosomal Storage disorders (https://www.esgld.org)
- WORLD Lysosomal Disease Network meetings (https://worldsymposia.org)
- Society for the Study of Inborn Errors of Metabolism (https://www.ssiem.org/)
- International Congress of Inborn Errors of Metabolism (http://www.iciem2017.org)
- American College of Medical Genetics (https://www.acmg.net)
- CNS annual meeting (https://www.cns.org/meetings/past-and-future-annual-meetings), American epilepsy Society meetings (https://meeting.aesnet.org)
- International Child Neurology conferences (https://www.epns.info/15th-international-child-neurology-congress)
- International League Against Epilepsy (<https://www.ilae.org>)
- International Bureau for Epilepsy (https://www.ibe-epilepsy.org)

## Appendix 2

*Expert Mapping Tool data analysis*.

Full mapping tool data has been presented in a separate abstract publication ([Poster](D:\\Documents\\Coufetery Comms\\Care beyond diagnosis\\CLN2 guideline manuscript\\Post Within3 manuscript 1 May 2020\\LDN Poster on CLN2 Guidelines Methodology.pdf)).

## Appendix 3

*Search criteria for the systematic literature review.*

- **MeSH terms**; ceroid lipofuscinosis-neuronal, late infantile ceroid lipofuscinosis, Batten Disease, Jansky-Bielschowsky.
- **Free text key words**; NCL, CLN2, LINCL, TPP1, clinical, aware*, diagnos*, therap*, retina OR ocular, counsel*, management (*Truncation symbol).

## Appendix 4

*PRISMA Flow diagrams*


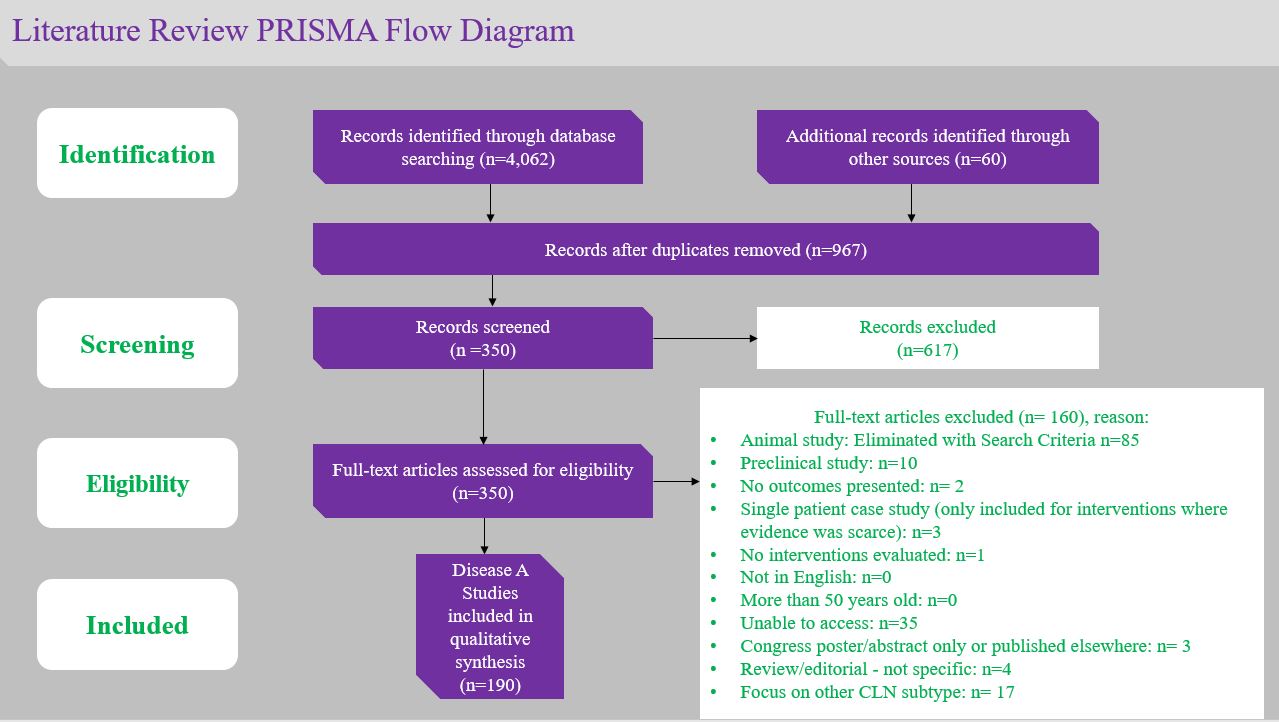


1. *Conducted by external medical writer*

##
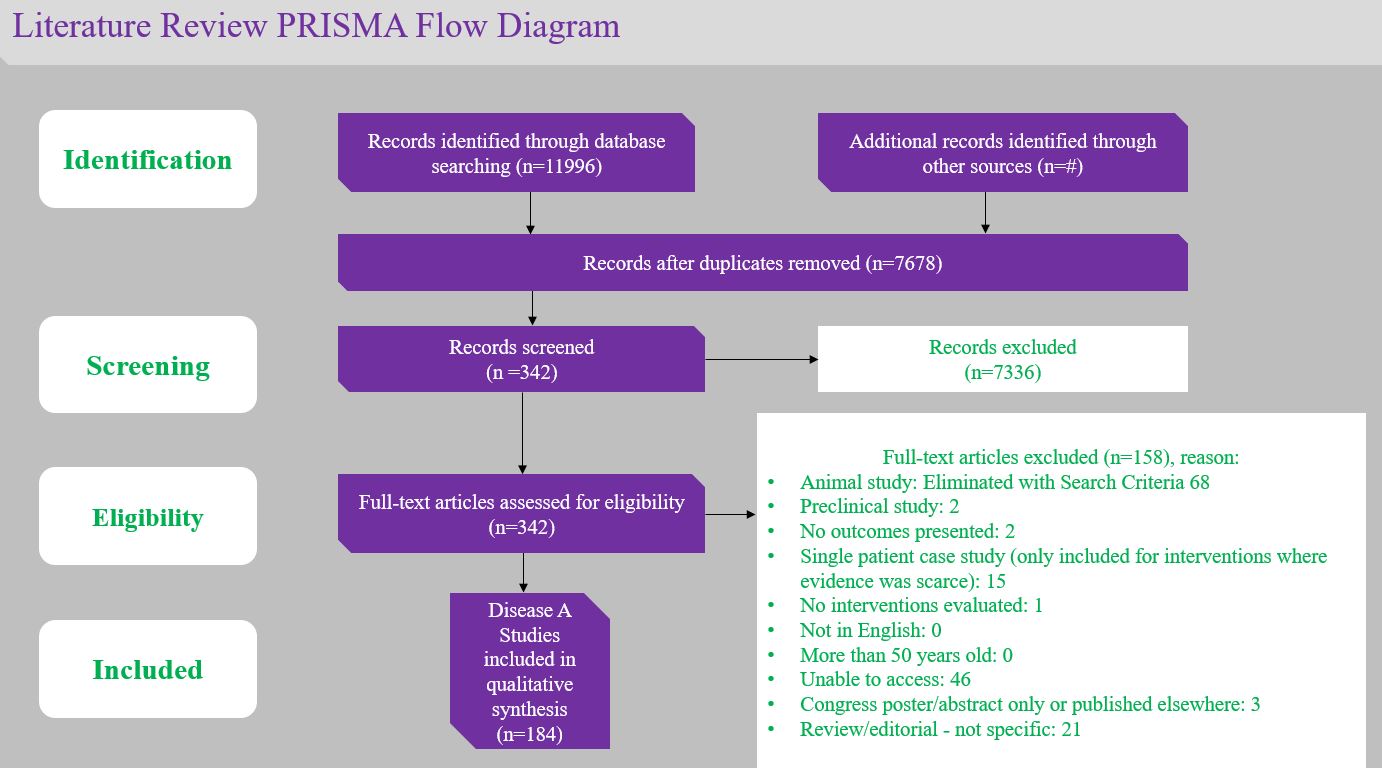


1. *Conducted by the CBD medical writer*

## Appendix 5

*Description of the Oxford Centre for Evidence-Based Medicine (OCEBM) criteria.*

Full details of the OCEBM can be found at the following link:

<https://www.cebm.net/2016/05/ocebm-levels-of-evidence/>

## Appendix 6

*Geographical location of the experts responding to the Modified Delphi questionnaire*.

The number of individuals from each country is either marked or is one individual. The graphical insert represents the areas of expertise.


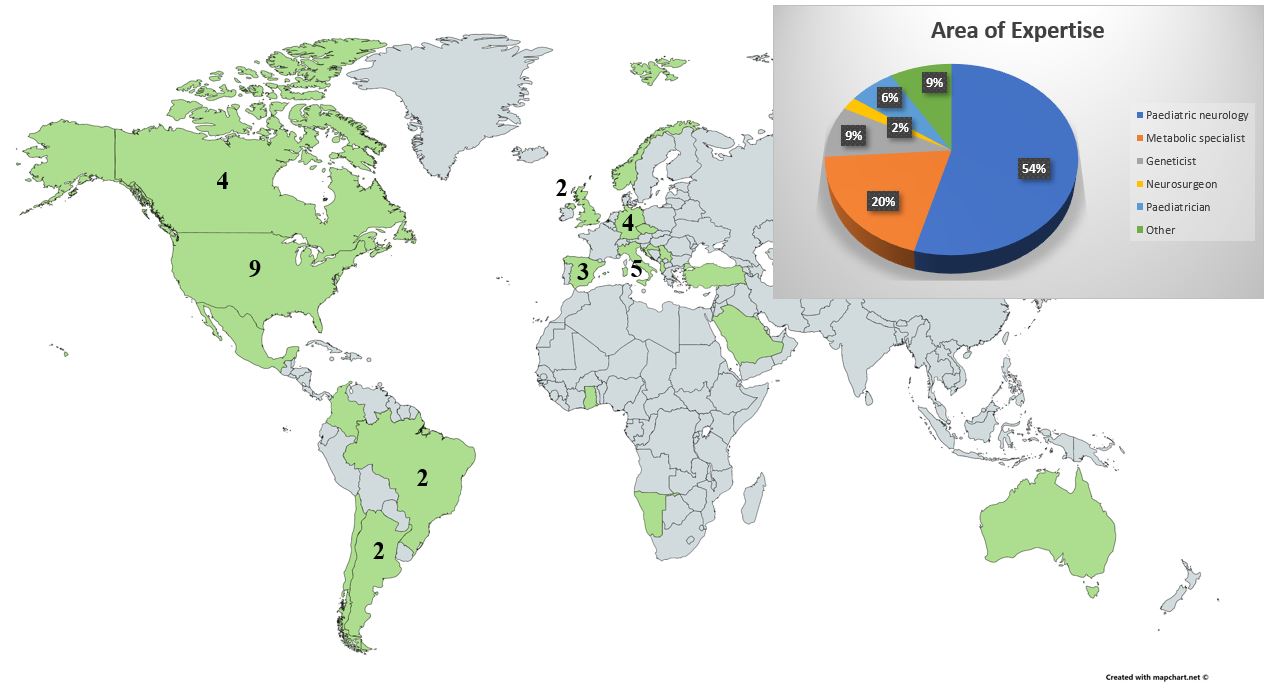


## Appendix 7

| **Domain** | **Question** | **Second review score**** | | **Total score** | **Minimum** | **Maximum** | ***(G-H) /(I)x100** | **Domain score** |  |  |  |  |  |  |  |  |  |
| --- | --- | --- | --- | --- | --- | --- | --- | --- | --- | --- | --- | --- | --- | --- | --- | --- | --- |
|  |  | **CF** | **RL** |  |  |  |  |  | *(obtained score-minimum possible score)/(maximum possible score) x 100. | | | | | | | | |
| Scope and Purpose | 1 | 7 | 7 | 14 | 2 | 14 | 85.71 | 83.3 |  |  |  |  |  |  |  |  |  |
|  | 2 | 7 | 7 | 14 | 2 | 14 | 85.71 |  |  |  |  |  |  |  |  |  |  |
|  | 3 | 7 | 6 | 13 | 2 | 14 | 78.57 |  | ** reversed score from that given to accurately reflect AGREE scoring system. | | | | | | | | |
| Stakeholder Involvement | 4 | 7 | 7 | 14 | 2 | 14 | 85.71 | 81.0 |  |  |  |  |  |  |  |  |  |
|  | 5 | 6 | 6 | 12 | 2 | 14 | 71.43 |  | Reviewers score | | 1 | 2 | 3 | 4 | 5 | 6 | 7 |
|  | 6 | 7 | 7 | 14 | 2 | 14 | 85.71 |  | True AGREE score | | 7 | 6 | 5 | 4 | 3 | 2 | 1 |
| Rigour of Development | 7 | 5 | 4 | 9 | 2 | 14 | 50.00 | 65.2 |  |  |  |  |  |  |  |  |  |
|  | 8 | 4 | 4 | 8 | 2 | 14 | 42.86 |  |  |  |  |  |  |  |  |  |  |
|  | 9 | 4 | 4 | 8 | 2 | 14 | 42.86 |  |  |  |  |  |  |  |  |  |  |
|  | 10 | 7 | 7 | 14 | 2 | 14 | 85.71 |  |  |  |  |  |  |  |  |  |  |
|  | 11 | 7 | 7 | 14 | 2 | 14 | 85.71 |  |  |  |  |  |  |  |  |  |  |
|  | 12 | 6 | 6 | 12 | 2 | 14 | 71.43 |  |  |  |  |  |  |  |  |  |  |
|  | 13 | 5 | 5 | 10 | 2 | 14 | 57.14 |  |  |  |  |  |  |  |  |  |  |
|  | 14 | 7 | 7 | 14 | 2 | 14 | 85.71 |  |  |  |  |  |  |  |  |  |  |
| Clarity of Presentation | 15 | 7 | 7 | 14 | 2 | 14 | 85.71 | 83.3 |  |  |  |  |  |  |  |  |  |
|  | 16 | 7 | 6 | 13 | 2 | 14 | 78.57 |  |  |  |  |  |  |  |  |  |  |
|  | 17 | 7 | 7 | 14 | 2 | 14 | 85.71 |  |  |  |  |  |  |  |  |  |  |
| Applicability | 18 | 6 | 6 | 12 | 2 | 14 | 71.43 | 50.0 |  |  |  |  |  |  |  |  |  |
|  | 19 | 6 | 6 | 12 | 2 | 14 | 71.43 |  |  |  |  |  |  |  |  |  |  |
|  | 20 | 1 | 1 | 2 | 2 | 14 | 0.00 |  |  |  |  |  |  |  |  |  |  |
|  | 21 | 5 | 5 | 10 | 2 | 14 | 57.14 |  |  |  |  |  |  |  |  |  |  |
| Editorial Independence | 22 | 7 | 6 | 13 | 2 | 14 | 78.57 | 78.6 |  |  |  |  |  |  |  |  |  |
|  | 23 | 7 | 6 | 13 | 2 | 14 | 78.57 |  |  |  |  |  |  |  |  |  |  |
| **Average total score** |  |  | **5.93** |  |  |  |  |  |  |  |  |  |  |  |  |  |  |

## Appendix 8

*Stop and start criteria sources for economic modelling*.

- England: (kindly supplied by Sheela Upadhyaya) National Institute for Health and Care Excellence (<https://www.nice.org.uk/guidance/indevelopment/gid-hst10008/documents>).
- France: Haute Autorité de Santé (HAS) Meeting on the 20^th^ June 2018 https://[www.has-sante.fr/portail/upload/docs/application/pdf/2019-03/brineura_summary_ct16359.pdf](https://www.has-sante.fr/portail/upload/docs/application/pdf/2019-03/brineura_summary_ct16359.pdf)
- Australia: Life Saving Drugs Programme (LSDP) (<http://www.health.gov.au/internet/main/publishing.nsf/Content/79DD3FACA8E75AE2CA257BF0001BAF7A/$File/batten-CLN2-guidelines-april-2019.PDF>)
- Canada: Canadian Agency for Drugs and Technologies in Health (CADTH) <https://www.cadth.ca/sites/default/files/cdr/complete/SR0574%20Brineura%20-%20Final%20CDEC%20Recommendation%20May%2027%2C%202019_for%20posting.pdf>

## Appendix 9

Lists of NCL support organisations can be found at this link: [https://www.ucl.ac.uk/ncl-disease/family-support](https://eur05.safelinks.protection.outlook.com/?url=https%3A%2F%2Fwww.ucl.ac.uk%2Fncl-disease%2Ffamily-support&data=02%7C01%7C%7C9a2c414eb72b453eb88408d7b93c7418%7C84df9e7fe9f640afb435aaaaaaaaaaaa%7C1%7C0%7C637181541600066956&sdata=Dk8URop2%2BXeAyFIDf10qq%2BcT%2FeAnx1FGXbyfHUKzlds%3D&reserved=0)

- **Batten Disease Family Association (BFDA)**

209-211 City Road, London EC1V 1JN. United Kingdom

**Phone:** 07876 682589

**Email:** admin@bdfa-uk.org.uk;

[www.bdfa-uk.org.uk](http://www.bdfa-uk.org.uk)

- **Batten Disease Support and Research Association (BDSRA)**

1175 Dublin Road, Columbus OH 43215, USA

**Phone:** 800-448-4570 (toll-free)

**Email:** info@bdsra.org

[www.bdsra.org](http://www.bdsra.org/)

- **Charlotte and Gwenyth Gray Foundation to Cure Batten Disease**

6033 West Century Boulevard, Suite 350, Los Angeles CA 90045, USA

**Phone:** 310-649-5222

**Email:** curebatten@givingback.org

[www.curebatten.org](http://curebatten.org/)

- **NCL Resource - A Gateway for Batten Disease**

MRC Laboratory for Molecular Cell Biology, University College London

Gower Street, London WC1E 6BT. United Kingdom

**Phone:** +00 44 207 679 7257

**Email:** ncl-www@ucl.ac.uk

[www.ucl.ac.uk/ncl](http://www.ucl.ac.uk/ncl)

- **Children's Brain Disease Foundation**

Parnassus Heights Medical Building, 350 Parnassus Avenue, Suite 900

San Francisco CA 94117, USA

**Phone:** 415-665-3003

**Fax:** 415-665-3003

**Email:** jrider6022@aol.com

- **Metabolic Support UK**

5 Hilliards Court, Sandpiper Way, Chester Business Park, Chester CH4 9QP.

United Kingdom

**Phone:** 0845 241 2173

**Email:** contact@metabolicsupportuk.org

[www.metabolicsupportuk.org](https://www.metabolicsupportuk.org/)

- **National Tay-Sachs and Allied Diseases Association, Inc. (NTSAD)**

2001 Beacon Street, Suite 204, Boston, MA, 02135, USA

**Phone:** 800-906-8723 (toll-free)

**Fax:** 617-277-0134

**Email:** info@ntsad.org

[www.ntsad.org](http://www.ntsad.org/)

- **Hope 4 Bridget** 904 Cortney Drive, Carpentersville, Illinois, 60110, USA davidkennicott@comcast.net
- **Noah’s Hope**  P.O. Box 54, Downers Grove IL 60515, USA [**https://www.noahshope.com/**](https://www.noahshope.com/)
- **A-NCL (Associazione Nazionale Ceroidolipofuscinosi)**

email address: [segreteria@a-ncl.it](mailto:segreteria@a-ncl.it)

web site: [www.a-ncl.it](https://eur03.safelinks.protection.outlook.com/?url=http%3A%2F%2Fwww.a-ncl.it%2F&data=02%7C01%7C%7C8c38f83797a247acb72508d7dad7dd6f%7C84df9e7fe9f640afb435aaaaaaaaaaaa%7C1%7C0%7C637218492971349579&sdata=pe8wU4lzdLQ%2FqbFCu7oKH7al0oGWq1UApulYg77TD0k%3D&reserved=0)

FaceBook account:  A-NCL Associazione Nazionale Ceroidilipofuscinosi Onlus

- **NCL-Gruppe Deutschland e.V. (German NCL Family Group)**

**Phone:** +49 30 411 26 19

**email:** Iris.Dyck@ncl-info.de

**web site:**https://www.ncl-deutschland.de

FaceBook account:  https://de-de.facebook.com/NCLDeutschland


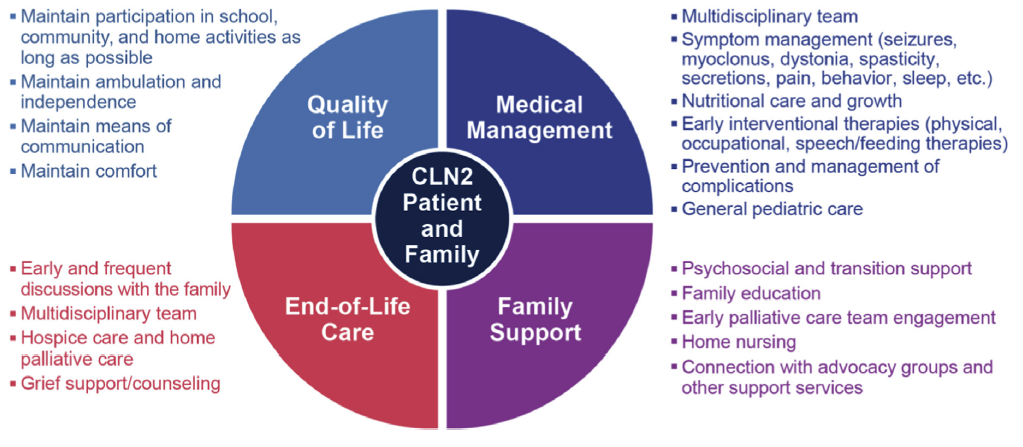


Figure 1.

A palliative care framework for CLN2 disease management facilitates comprehensive care of patients and families. Figure taken from Williams R. E et al., (2017)  Management strategies for CLN2 disease. <http://dx.doi.org/10.1016/j.pediatrneurol.2017.01.034>. Published by Elsevier Inc. an open access article under the CC BY-NC-ND license (<http://creativecommons.org/licenses/by-nc-nd/4.0/>).
