# Supplementary material for: Guidelines on the diagnosis, clinical assessments, treatment and management for CLN2 disease patients
Source: Orphanet J Rare Dis. 2021 Apr 21;16:185. doi: 10.1186/s13023-021-01813-5 (PMC8059011; doi:10.1186/s13023-021-01813-5)
Supplement: Supplementary file 2 — Additional file 2: Poster. Methodology to develop guidelines for the management of patients with CLN2 disease. [file 13023_2021_1813_MOESM2_ESM.pdf]

Methodology To Develop Guidelines For The Management Of Patients With Neuronal Ceroid Lipofuscinosis Type 2 Disease

Authors:
Hendriksz CH[1], Donohue J[2], Donohue Y[2], Mole SE[3]
Author Information:
1. University of Pretoria, Steve Biko Academic Unit, Pretoria, South Africa
2. Care Beyond Diagnosis, Columbia, TN, US
3. MRC Laboratory for Molecular Cell Biology, UCL Institute of Child Health, University College London, London, UK.

Care Beyond Diagnosis
US, Non-Profit Organization: Tax ID : 38-4040391

The multiple clinical manifestations and progressive nature of CLN2 disease, a major form of the neuronal ceroid lipofuscinoses or Batten disease, make the management of patients challenging. Although guidelines are available, the methodology used to formulate clinical recommendations has come under increased scrutiny, highlighting a need for robust, independent guidance on the benefits/risks of disease-modifying treatments and the medical interventions used to manage this condition. This method will be implemented to develop evidence-based and expert-agreed recommendations for the wide range of interventions currently used to manage CLN2 disease and will be independently managed by Care Beyond Diagnosis, a 501(c)3.

The first part of the project was to develop an expert mapping tool to identify two chairs, who would lead the project to ensure the process is transparent and unbiased. After identifying 1,454 professionals through the KOL tool, after ranking by the "tool", they were sequentially approached until two were able to commit to participation in this project.

Key statements or questions will be developed and supported by a systematic review of the published evidence using PRISMA guidance to form the basis of an international modified Delphi consensus determination process which will then be conducted.

Multiple stakeholders have been identified and approached to support funding this project, including; potential companies developing therapeutics in this area, patient organisations and an international steering committee as recommended by the steering committee chairs.
The guidelines will be assessed against the Appraisal of Guidelines for Research and Evaluation (AGREEII) criteria and multiple tools will be developed to support their implementation and after completion, a written report will be produced.

This approach has been used successfully for development of other rare disorders for example ACMG PKU and the MSUD Guidelines, which led to publication.

CLN2 Management Guidelines Overview

EXPLORATION
- Program initiation
- Run KOL Mapping
- Consider and invitation of Stakeholders
- Contract in 2 Chairs of SC
- Preliminary review of Evidence
- Identify Scope through Introductory call
- Identify & Confirm Bib. Fellows
- Chairs to identify up to 30 experts
- Submit Abstract
- Open Consensus Platform
- Completion of Literature Review
- Draft statements/questions

CONSENSUS BUILDING
- First Meeting Virtually
- Update list of questions/statements
- Begin Delphi-1
- Second meeting preferably F2F
- Update list of questions/statements
- Run Delphi 2
- IF…
- 3rd virtual Meeting if necessary
- Run Delphi 3
- Update the Literature Review
- CONSENSUS

COMMUNICATING THE CONSENSUS
- Complete and submit the Manuscript
- National Guidelines Clearinghouse or SIGN Assessment
- Share Publication
- Launch Guidelines
- Begin the development and promotion of the Implementation Tools

Q1	Q2	Q3	Q4	Q1	Q2	Q3	Q4	Q1
2018				2019				2020

Guidelines Assessment Criteria

Table 1. Notes on use of the AGREE II instrument for guideline quality evaluation in rare diseases

AGREE II Domain	Points to consider
Scope and purpose (Items 1–3)	Rare disease guidelines should be able to address all of the items concerned with scope and purpose.
Stakeholder involvement (Items 4–6)	Although it is likely that one professional group may dominate, comprehensive stakeholder involvement is as important to the development of guidelines for rare diseases as it is for common diseases. Scoring of these items should recognise this principle and reflect the extent to which the guideline addresses each item.
Rigour of development (Items 7–14)	The AGREE II quality rating does not depend on the quantity or type of published evidence but on the rigour of the systematic methods used to identify, select and synthesize evidence and the transparency with which the guideline development group report how they reached recommendations. For item 13 (external review by experts) – the experts should include patients, carers, and/or patient groups.
Clarity of presentation (Items 15–17)	When scoring item 16 there may not be a range of options for management of the (rare) condition or health issue. In this case the item would be considered 'not applicable' and scored as '1'.
Applicability (Items 18–21)	The extent to which a guideline can provide information on potential facilitators to guideline implementation and describe resource implications may be limited for rare disease guidelines where the implementation setting is likely to encompass diverse healthcare contexts. The information provided may be country-specific, healthcare-system-specific, or generic
Editorial independence (Items 22–23)	For many rare diseases there are likely to be only a small number of experts worldwide. This may limit the potential for editorial independence. Scores should reflect how this was addressed.
Overall guideline assessment	Before selecting 'yes with modifications', consider whether resources are available to modify the guideline and any copyright issues. The existence of only a few or only one guideline on a topic should not prevent a judgment of 'no' on question 2 as it is worthwhile to indicate that better quality guidelines are needed.
Notes section	Indicate if the guideline is the only (known) guideline on available on the topic. Indicate any research recommendations which the guideline identifies.

Identifying your Key Opinion Leaders without perceived "bias" utilizing novel Expert Mapping tool

1.) Research and Grading of Experts

CLN2 Experts were allocated points based on their publication history, patient group involvement and scientific event engagement

2.) Categorize, Exclude and Engagement of highest ranked experts for Steering Committee Co-Chair (X2) Selection

Once the list had been ranked, only clinicians were considered for Chair positions along with their geographic location permitting 'one' expert from Europe and 'one' from any country outside of Europe to drive international collaboration

3.) Co-Chairs provide feedback on SC Experts

Steering Committee members should be of well balanced backgrounds including a working history of CLN2 Treatment

Co-Chairs should also consider highly those with high involvement in Patient Advocacy Groups and MDT Representation

4.) Engage the Steering Committee Prospects

Tools
- PubMed literature search
- SCOPUS H-Index Review
- Patient Organization Event Search of last 'five' Years
- Scientific Conference/Event search of last 'five' Years

Method:	Weighted Score	
A search of PubMed for all relevant articles to CLN2	2<br>3 - 5<br>6 - 9<br>10+	1<br>2<br>4<br>5
Using SCOPUS Database, Record H-Index of Author	>30<br><30	3<br>6
Involvement at Patient Organization Events as Speaker or Chair	>3<br><3	6<br>9
Involvement at Scientific Congress/Events	2-5<br>6-9<br><9	2<br>4<br>6

Steps A, B, C, and D Details and Outcomes

A. Literature Search
1. PubMed search " CLN2 and Batten disease"
2. Filtered for "Clinical trial, meta-analysis, systematic review and review"
3. Last 20 Years Only
4. Identified CLN2-related all authors of articles
5. Hand-screening to remove irrelevant publications and identify relative experience in CLN2
6. Allocate score according to scale
7. *Only include authors which were on 'two' or more publications.

RESULTS: 155 "Relevant" Publications; 717 Published Authors; 124 Authors Scored*

B. Scopus Search
1. Search authors by last name and first name to reveal H-Index
2. Find the profile based on occupation, middle initial and city/country of residence.
3. Allocate score according to scale.

RESULTS: 124 Authors Scored*; Add step 1 with step 2 and re-allocate points

C. Patient Organisation Events
1. Search for all conferences in the past **5 years** with Google and Internet explorer with term CLN2, Batten Disease and Neuronal Ceroid Lipofuscinosis.
2. Record all the chairs, Poster Contributors and oral presenters.
3. Cross reference BDFA and BDSRA, along with other CLN2 focused organizations
4. Record all oral presentations, poster presentations and chairs
5. Allocate score based on the scale.

RESULTS: '7' Events with programs; 184 Speakers, chairs and contributors

D. Scientific Events
1. Search all the programs for the following scientific conferences and record all chairs and oral presenters in past 5 years.
2. Scientific relevant events- European Paediatric Neurology Society, NCL International Conferences, European Study group for Lysosomal Storage disorders, WORLD Lysosomal Disease Network meetings, SSIEM, ICIEM, ACMG, CNS annual meeting, American epilepsy Society meetings, International Child Neurology conferences, ILAE/IBE events.
3. Record all chairs and oral presentations
4. *Narrow list by counting only those with 2x appearances

RESULTS: 34 Events Identified; 1,731 Individuals Identified; 4*. List narrowed to 494 Experts

Criteria

- a Clinician whom works directly with CLN2 patients*
- Retired clinicians will be considered
- Must include '1' clinician as chair from Europe, and '1' from the rest of the world
- Animal Experts excluded from consideration for Chair
- Industry professionals excluded

Invite experts to join as a co-chair in order of their scoring, excluding only for reasons listed here, making note of any exclusion or expert's decision not to participate

Criteria created in an effort to identify experts with the most on-hands patient experience, remove bias and to be geographically balanced.

Exception to Criteria: process to run through leading clinicians and once leading clinicians with geographic contrast are exhausted, all experts on list to be reconsidered in order of total score: regardless of geographical separation or clinical experience.

- **1454** Total Candidates identified through process
- Highest and lowest individual scores: '1' Low--- '21' High
- Exclusions were made, and list was populated
- 2 Chairs selected with individual scores of 20 and 16

Grading Existing Literature

Table 2. OCEBM Levels of Evidence Working Group*. "The Oxford 2011 Levels of Evidence".

Question	Step 1<br>Level 1	Step 2<br>Level 2	Step 3<br>Level 3	Step 4<br>Level 4	Step 5<br>Level 5
How common is the problem?	Local and current random sample surveys (or censuses)	Systematic review of surveys that allow matching to local circumstances**	Local non-random sample**	Case-Series**	n/a
Is this diagnostic or monitoring test accurate? (Diagnosis)	Systematic review of cross sectional studies with consistently applied reference standard and blinding	Individual cross sectional studies with consistently applied reference standard and blinding	Non-consecutive studies, or studies without consistently applied reference standards**	Case-control studies, or "poor or non-independent reference standard**	Mechanism-based reasoning
What will happen if we do not add a therapy? (prognosis)	Systematic review of inception cohort studies	Inception cohort studies	Cohort study or control arm of randomized trial*	Case-series or case-control studies, or poor quality prognostic cohort study**	n/a
Does this intervention help? (treatment benefits)	Systematic review of randomized trials or n- of-1 trials	Randomized trial or observational study with dramatic effect	Non-randomized controlled cohort/follow-up study**	Case-Series case-control studies, or historically controlled studies**	Mechanism-based reasoning
What are the common harms? (Treatment harms)	Systematic review of randomized trials, systematic review of nested case control studies, n-of-1 trial with the patient you are raising the question about, or observational study with dramatic effect	Individual randomized trial or (exceptionally) observational study with dramatic effect	Non-randomized controlled cohort/follow-up study (post-marketing surveillance) provided there are sufficient numbers to rule out a common harm. (Fr long-term harms the duration of follow-up must be sufficient.)**	Case-series, case control, or historically controlled studies**	Mechanism-based reasoning
What are the RARE harms? (Treatment harms)	Systematic review of randomized trials or n-of-1 trial	Randomized trial or (exceptionally) observational studies with dramatic effect			
Is this (early detection) test worthwhile? (screening)	Systematic review of randomized trials	Randomized trial	Non-randomized controlled cohort/follow-up study**	Case-series, case-control, or historically controlled studies**	Mechanism-based reasoning

Scope
"To provide guidance on reliable diagnosis, clinical assessments, availability of therapies, and disorder management for CLN2 patients of any age."

Achieving Consensus
During all meeting all meetings experts review and vote on all aspects of guidelines and only those considered to reach a group consensus are to be added to the manuscripts.

Accreditation
To maximize the impact of the guidelines, the final project will be assessed against the AGREEII criteria and given a score out of a 7. 6 and higher are considered to be an exceptional set of clinical guidelines.

Outcomes
Patients can assist clinicians in selecting best patient-centered care.
Increase diagnosis rate and management inline with best available evidence.
Supporting non experts in managing patients.
Unbiased opinions for funders.

Dissemination
Medical Journal Publication to be shared through partnering patient organizations and clinical networks. Utilize networks of clinicians and other with commitment in this field.

Table 1.) Hilton Boon M, Ritchie K, Manson J. Improving the retrieval and dissemination of rare disease guidelines and research recommendations: a RARE-Best Practices initiative. Rare Dis Orphan Drugs 2014;1(1):20-9
Table 2.) OCEBM Levels of Evidence Working Group*. "The Oxford 2011 Levels of Evidence". Oxford Centre for Evidence-Based Medicine. http://www.cebm.net/index.aspx?o=5653 * OCEBM Table of Evidence Working Group = Jeremy Howick, Iain Chalmers (James Lind Library), Paul Glasziou, Trish Greenhalgh, Carl Heneghan, Alessandro Liberati, Ivan Moschetti, Bob Phillips, Hazel Thornton, Olive Goddard and Mary Hodgkinson
